# An Increase of Adropin Can Predict Depression Improvement

**DOI:** 10.3390/nu17101666

**Published:** 2025-05-14

**Authors:** Duška Krnić, Sara Sablić, Maja Marinović Guić, Danijela Budimir Mršić, Dragan Krnić, Romilda Roje, Daniela Šupe Domić, Sanja Lovrić Kojundžić

**Affiliations:** 1 Department of Psychiatry, University Hospital of Split, 21000 Split, Croatia; krnic.duska@gmail.com (D.K.); rojeromilda@gmail.com (R.R.); 2 Clinical Department of Diagnostic and Interventional Radiology, University Hospital of Split, 21000 Split, Croatia; sarasablic@gmail.com (S.S.); maja.marinovic.guic@gmail.com (M.M.G.); danijelabudimir@gmail.com (D.B.M.); 3 School of Medicine, University of Split, 21000 Split, Croatia; drkrnic@gmail.com; 4 University Department of Health Studies, University of Split, 21000 Split, Croatia; daniela.supedomic@gmail.com; 5 Department of Surgery, University Hospital of Split, 21000 Split, Croatia; 6 Department of Medical Laboratory Diagnostics, University Hospital of Split, 21000 Split, Croatia

**Keywords:** adropin, depression, vitamin D, inflammation

## Abstract

Objectives Depression is characterized by a lack of energy, social withdrawal, and fatigue, and it is also associated with increased inflammation in the brain. Some studies suggest that adropin may have anti-inflammatory effects and could reduce the inflammatory processes contributing to depression. Methods: We included 54 newly diagnosed patients experiencing their first episode of depression and 56 healthy volunteers in this study. The participants with depression were divided into three subgroups based on DSM-5 and BDI-II criteria. The focus of the study was to compare adropin levels between depressive patients and healthy volunteers, as well as to monitor changes in adropin levels after six months of treatment for depressive patients. Results: Initial measurements showed no significant differences in standard laboratory parameters or adropin levels between the depression and control groups. However, adropin and vitamin D levels increased in the group of depressive patients during the six-month follow-up. Conclusions: Our research indicates that adropin plays a significant role in the development of depression and may influence the effectiveness of depression treatments.

## 1. Introduction

Since the discovery of adropin as a regulatory hormone in 2008, numerous studies have revealed its beneficial role in human organisms, particularly regarding metabolism and energy homeostasis [1]. Adropin is encoded by the energy homeostasis-associated gene (Enho) and is primarily expressed in the liver and brain. It is also present in other tissues such as the heart, lung, renal medulla, muscles, peripheral blood, and breast cancer cells [1,2,3]. Adropin has been further investigated in the context of sex differences, which proved that women had lower serum adropin values than men [4]. Moreover, one study showed young, thin men to have the highest adropin levels [5]. It was also demonstrated that, in contrast to women, men’s levels of circulating adropin were inversely correlated with their levels of low-density lipoprotein cholesterol (LDL-C) [5,6], which partially explained the role of adropin in the atherosclerosis process. Decreased adropin levels were found in patients with coronary heart disease (CHD), acute myocardial infarction, and hypertension [7,8,9], but also in obese patients and adults with low physical activity [8,10]. While much of the above-mentioned research on adropin has focused on metabolic and cardiovascular disorders, there is an increasing interest in its potential role in neurologic, especially in psychiatric diseases, such as depression. Depression is one of the most common emerging new psychiatric mood disorders in the world, and, according to reports, 17% of people will experience depression at some point in their lives [11].

As far as we know, depression has been associated with increased inflammation in the brain [12]. Some studies have suggested that adropin may have anti-inflammatory effects, which could reduce the inflammatory processes that contribute to depression [13]. According to recent research, adropin has neuroprotective properties, and its low levels may relate to adropin-related depressive-like behaviors [14]. Furthermore, the deregulation of adropin in patients with depression could have contributed to the metabolic abnormalities observed in depression [5]. Also, adropin may play a role in decreasing low-grade inflammation, which could modulate depression-related inflammation [15]. Studies additionally revealed cellular and molecular changes in the depressed brain, as well as anomalies in the brain circuitry [16,17]. The pathophysiology of neuropsychiatric disorders, including depression, is linked to a disrupted serine/threonine kinase glycogen synthase kinase 3β (Akt/GSK3β signaling pathway) [18,19]. According to research with mice fed a high-fat diet, the consequential obesity induced desensitizing of serotonin-dependent Akt/GSK3β signaling and caused depression. Therefore, improving Akt/GSK3β inhibitory control is an essential component of treatment for depressive disorders [18].

In a study of coronary heart disease (CHD) patients comorbid with depression, the serum levels of irisin, adropin, preptin, and brain-derived neurotrophic factor (BDNF) were significantly reduced in both CHD patients with depression and those without depression [7]. These studies revealed associations between energy homeostasis and CHD, particularly between depression and blood levels of adropin, preptin, irisin, and brain-derived neurotrophic factor (BDNF).

Adropin can activate the ERK 1/2 signaling pathway via VEGFR-2, influencing BDNF—known for its neuroprotective properties essential to neurogenesis, synaptic formation, and memory [20]. Despite these insights, there is a lack of comprehensive studies on adropin levels in depressed individuals. Our study aims to address this gap by comparing adropin levels in newly diagnosed depression patients with matched healthy controls and assessing changes over a six-month treatment period. This research could significantly enhance our understanding of adropin’s role in managing depression.

## 2. Materials and Methods

### 2.1. Study Design and Study Population

This prospective study was performed at the Psychiatry Department, University Hospital of Split, from September 2022 to September 2023. It was carried out in accordance with the Helsinki Declaration and approved by the Ethics Committee of the University Hospital of Split on 05.09.2022. (Nr. 2181-147/01/06/M.S.-22-2, Class: 500-03/22-01/71). All subjects signed a written informed consent form before participating in the study.

The study population consisted of 56 newly diagnosed adult patients with depression, all aged 18 years or older, who were experiencing their first depressive episode (referred to as the depressive disorder group—DDG) according to the DSM-5 (Diagnostic and Statistical Manual of Mental Disorders, Fifth Edition). The control group included 54 subjects who were matched by gender and age and who had no major illnesses or mental disorders.

Both groups completed the Beck Depression Inventory-II (BDI-II), with a cutoff score of 14 or greater used to diagnose depression. Volunteers were recruited from the University Hospital of Split’s Department of Medical Laboratory Diagnostics to participate as healthy controls, with no compensation provided.

Exclusion criteria were identical for both study groups and included chronic inflammatory or metabolic diseases, hypertension, peripheral vascular or obstructive pulmonary disease, renal failure, pregnancy, thyroid disorders, the use of certain medications (such as hypnotics, sedatives, and immunosuppressants), or following a prescribed special diet for any reason. All subjects completed an informed consent form along with a questionnaire regarding their health status and previous illnesses.

### 2.2. Clinical and Laboratory Parameters

The study participants passed a comprehensive assessment, including taking a detailed medical history and physical examination, including calculation of body mass index (BMI) according to the formula: weight (kg) divided by height squared (m^2^). A short questionnaire made by investigators was used for the assessment of physical activity and specific addiction to cigarettes, alcohol, or any other psychostimulants.

According to the standard laboratory procedures, we measured these laboratory parameters in both groups: total blood count, C-reactive protein (CRP; mg/L), glucose (mmol/L), triglycerides (mmol/L), total cholesterol (mmol/L), low-density lipoprotein cholesterol (LDL; mmol/L), hemoglobin A1c (mmol/L), unsaturated iron binding capacity (UIBC, mcg/dL), and total iron binding capacity (TIBC, mcg/dL). The additional laboratory analysis included measuring some vitamin and hormone serum levels using an immunoassay analyzer (“ECLIA” Roche Diagnostics, Penzberg, Germany). These include vitamin B12 (pg/mL), folate (nmol/mL), vitamin D (nmol/mL), thyroid stimulating hormone (TSH, mIU/L), T3 (nmol/L), and T4 (nmol/L).

### 2.3. Adropin Analysis

According to the standard laboratory procedure, the same experienced biochemist analyzed the adropin serum levels in both groups using a commercially available enzyme-linked immunosorbent assay test (Roche Diagnostics GmbH, Mannheim, Germany). Fasting venous blood samples were taken and placed in ethylenediaminetetraacetic acid (EDTA) tubes. After centrifugation, plasma was frozen and stored at −80 °C for further adropin analyses. The first blood samples were taken at admission (before the specific therapy) for the determination of baseline adropin levels. The control blood samples from the study group were taken six months after the beginning of treatment.

### 2.4. Assessment of Depression Score

The diagnosis of depression was established by a psychiatrist through a structured psychiatric interview, in accordance with the diagnostic criteria outlined in the DSM-5. The control group was likewise selected by the psychiatrist using the same interview method and DSM-5 guidelines to confirm the absence of depressive symptoms. Both groups answered the 21-item BDI-II, a commonly used questionnaire measuring the severity of depression in adolescents and adults [21]. Each item is scored on a four-point scale from 0 to 3, with possible total BDI-II scores ranging from 0 to 63. Depression was classified into four categories based on the BDI-II scores: no depression or slight depression (0–13), mild depression (14–19), moderate depression (20–28), and severe depression (29–63). BDI-II was evaluated after confirming a depression diagnosis before any medical therapy and reassessed following the six-month treatment period.

### 2.5. Patient Follow-Up

The 54 newly diagnosed patients were all routinely monitored by the same doctor and treated with escitalopram (a selective serotonin reuptake inhibitor antidepressant) along with additional benzodiazepines if needed for anxiety or sleep issues. In addition to retaking the 21-item BDI-II exam and having their blood samples taken for serum adropin levels, all patients received the same mental evaluation at the 6-month follow-up (reevaluation in accordance with the DSM-5).

### 2.6. Statistical Analysis

Statistical Package for the Social Sciences—SPSS software (version 20 for Windows; SPSS Inc.; Chicago, IL, USA) was used for statistical analysis. Qualitative variables were described as absolute and relative values. Median and interquartile values were used to describe quantitative variables. The significance of differences in qualitative characteristics was calculated by the c^2^ test as well as logistic regression. Analysis of the statistical significance of differences in quantitative variables was performed by the Mann–Whitney U test or by the Wilcoxon test. Statistical significance was set up to *p* < 0.05, and all confidence intervals were given at the 95% level.

## 3. Results

The study included 110 patients, 54 (49%) patients within the depressive disorder group (DD group) and 56 (51%) patients within the control group. The DD group and control groups did not differ significantly in demographic and clinical data, as shown in Table 1. Women were significantly more numerous, accounting for more than 80% in both groups. The median age of the study population was 45.5 years, with no age difference between sexes (45 years for men vs. 45.5 years for women, *p* = 0.634). Other characteristics, such as BMI, specific addictions, and menopausal state, showed no difference between the groups. The control group reported no evidence of depression.

In the DD group, the most common form of depression was severe depression in 54% of patients, while mild and moderate forms were less common (24% and 22%, respectively) (Table 1).

No significant difference between the DD and control groups regarding following laboratory parameters was found: leukocytes (Z = 0.191; *p* = 0.848), blood glucose (Z = 1.08; *p* = 0.281), CK (Z = 0.427; *p* = 0.669), cholesterol (Z = 0.408; *p* = 0.683), triglycerides (Z = 0.680; *p* = 0.497), HDL (Z = 0.151; *p* = 0.880), LDL (Z = 0.658; *p* = 0.510), Fe (Z = 0.828; *p* = 0.408),TIBC (Z = 1.2; *p* = 0.246), vitamin B12 (Z = 1.3; *p* = 0.204), vitamin D1 (Z = 0.112; *p* = 0.911), T4 (Z = 0.075; *p* = 0.940), TSH (Z = 1.0; *p* = 0.314), adropin (Z = 0.918; *p* = 0.359), CRP (Z = 1.9; *p* = 0.061), UIBC (Z = 1.7; *p* = 0.090), transferrin saturation (Z = 1.8; *p* = 0.078), and folic acid levels (Z = 1.71; *p* = 0.088) (Table 2).

Only the level of thyroid hormone T3 was higher in the depression group than in the control group (Z = 2.5; *p* = 0.013).

The Beck Depression Inventory II was completed twice during the study period: on initial evaluation (baseline BDI-II) and six months following the beginning of therapy. During this follow-up period of 13 patients categorized as having mild depression, 11 were in remission after treatment, 1 remained mildly depressed, and 1 worsened to moderate depression. Of nine patients categorized as having moderate depression, six were in remission after treatment, two turned into mild forms of depression, and one remained in the same category of moderate depression. The majority, 28 of them, had severe depression at the beginning, of which 15 patients were in remission after treatment, 6 changed to mild depression, 5 changed to moderate depression, and 2 remained the same. This implies that after the treatment of 50 depressive patients, 32 (64%) were successfully treated and in remission, and 13 (26%) improved to milder forms—these patients belong to responders. The remaining participants were non-responders: four patients (8%) in the DD group remained unchanged, and one (2%) worsened during the therapy (Table 3).

No significant difference was found between the two samplings (beginning of study and six-month follow-up) in blood glucose (Z = 0.269; *p* = 0.788), CK (Z = 215; *p* = 830), CRP (Z = 1.24; *p* = 0.216), cholesterol (Z = 0.626; *p* = 0.532), triglycerides (Z = 1.45; *p* = 0.147), HDL (Z = 0.912; *p* = 0.363), LDL (Z = 0.285; *p* = 0.776), Fe (Z = 0.127; *p* = 0.899), UIBC (Z = 0.971; *p* = 0.332), TIBC (Z = 0.454; *p* = 0.650), transferrin saturation (Z = 0.313; *p* = 0.754), vitamin B12 (Z = 1.33; *p* = 0.184), folic acid (Z = 1.0; *p* = 0.315), T4 (Z = 0.190; *p* = 0.849), and T3 levels (z = 1.23; *p* = 0.218) (Table 4).

More precisely, the median vitamin D1 was 7.7 (95%CI: −17.5 to 2.1) higher in the second sampling than in the first one (Z = 2.12; *p* = 0.034). More precisely, in 19 (38%) patients there was a decrease in vitamin D1, in 30 (60%) there was an increase in vitamin D1, and in one patient the value remained the same. The median adropin level was 0.17 (95%CI; −0.55 to 0.21) higher in the second sampling than in the first sampling (Z = 2.37; *p* = 0.018). In 12 (24%) patients there was a fall in adropin levels, and in 38 (76%) there was an increase.

In the subgroup of responders (N = 32) there was no statistically significant difference between the first and second sampling in the levels of leukocytes (Z = 1.11; *p* = 0.266), blood glucose (Z = 0.453; *p* = 0.650), CK (Z = 0.911; *p* = 0.362), CRP (Z = 0.944; *p* = 0.345), cholesterol (Z = 0.283; *p* = 0.777), triglycerides (Z = 1.26; *p* = 0.206), HDL (Z = 0.979; *p* = 0.328), LDL (Z = 0.181; *p* = 0.856), Fe (Z = 0.039; *p* = 0.969), UIBC (Z = 0.852; *p* = 0.394), TIBC (Z = 0.944; *p* = 0.345), transferrin saturation (Z = 0.122; *p* = 0.903), folic acid (Z = 0.402; *p* = 0.688), vitamin D (Z = 1.64; *p* = 0.102), vitamin B12 (Z = 1.7; *p* = 0.085), T4 (Z = 0.039; *p* = 0.969), and T3 (Z = 0.833; *p* = 0.405) (Table 5). The median of the second sampling of adropin was higher by 3.36 than in the first sampling (Z = 3.5; *p* = 0.001). Adropin increased in 28 (87%) and decreased in only 4 responders (13%).

In the subgroup of non-responders (N = 18), there was no significant difference between the first and second sampling of leukocytes. In the subgroup of non-responders (N = 18), there was no significant difference between the first and second sampling of leukocytes (Z = 1.42; *p* = 0.155), blood glucose (Z = 1.26; *p* = 0.206), CK (Z = 1.02; *p* = 0.309), CRP (Z = 1.102; *p* = 0.306), cholesterol (Z = 0.594; *p* = 0.553), triglycerides (Z = 0.767; *p* = 0.443), HDL (Z = 0.061; *p* = 0.951), LDL (Z = 0.356; *p* = 0.722), Fe (Z = 0.492; *p* = 0.623), UIBC (Z = 0.426; *p* = 0.670), TIBC (Z = 0.626; *p* = 0.531), transferrin saturation (Z = 0.466; *p* = 0.641), vitamin B12 (Z = 0.071; *p* = 0.943), folic acid (Z = 1.16; *p* = 0.246), vitamin D (Z = 1.2; *p* = 0.227), T4 (Z = 0.095; *p* =0.925), T3 (Z = 1.03; *p* = 0.301), nor adropin levels (Z = 0.109; *p* = 0.913).

The change in laboratory parameters after treatment was not statistically significantly different between the responders (n = 32) compared to non-responders (n = 18) regarding the leukocytes (Z = 1.3; *p* = 0.192), CRP (Z = 1.1; *p* = 0.273), cholesterol (Z = 0.414; *p* = 0.679), triglycerides (Z = 0.802; *p* = 0.422), HDL (Z = 0.282; *p* = 0.778), LDL (Z = 1.1; *p* = 0.283), Fe (Z = 0.670; *p* = 0.503), UIBC (Z = 0.537; *p* = 0.591), TIBC (Z = 0.141; *p* = 0.888), transferrin saturations (Z = 0.423; *p* = 0.672), folic acid (Z = 1.4; *p* = 0.162), vitamin D (Z = 1.03; *p* = 0.303), T4 (Z = 0.863; *p* = 0.388), and T3 levels (Z = 1.28); *p* = 0.202) (Table 6).

**Figure 1:**
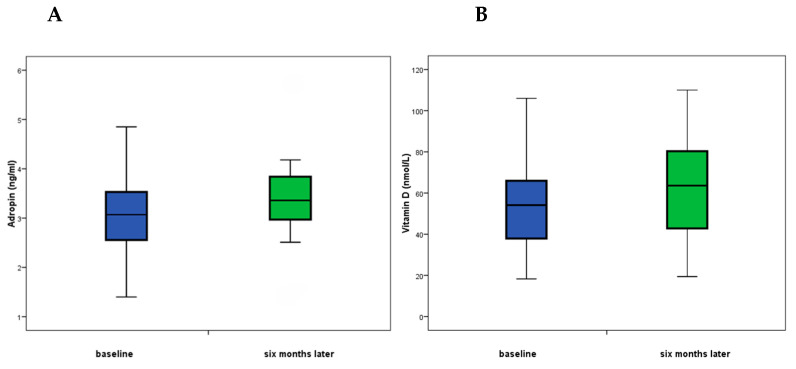
(**A**) Serum adropin levels in responders at baseline and after the six-month follow-up; (**B**) vitamin D levels in responders at baseline and after the six-month follow-up. Data are presented as medians.

Changes after treatment show that adropin in non-responders was lower in the second measurement than in the first measurement, which was significantly different compared to the responders, in whom adropin increased (Z = 1.98; *p* = 0.048), Figure 1. Similarly, the difference between first and second blood glucose measurements was higher in the non-responders; the median difference was 0.25, compared to a change of 0.05 in the responders (Z = 2.0; *p* = 0.45). The change between the first and second CK measurements showed that the median decrease in muscle activity in non-responders was 18, and the increase in muscle activity in responders was 2.5 (Z = 2.2; *p* = 0.027). The median change between baseline and second vitamin B12 measurements in non-responders was 21, and in responders was 32.5 (Z = 2.2; *p* = 0.032) (Table 6).

## 4. Discussion

The purpose of our study was to compare the levels of the regulating hormone adropin and laboratory measures in patients with recently diagnosed depressive disorders to those of healthy control-matched individuals at the time of diagnosis and over a 6-month follow-up period. The most important finding was that adropin levels increased in a group of depressive patients who were successfully treated (the responders).

Our results indicate that adropin levels in patients with depressive disorders do not significantly differ from those in the control group at the outset. This finding contrasts with some previous studies that analyzed adropin levels in depressed patients, who were primarily diagnosed with chronic depression [7,15]. Additionally, we did not observe significant differences in other tested laboratory parameters. One possible explanation for these contrasting results regarding initial adropin levels could be that the patients in earlier studies were notably older, predominantly male, and had significant comorbidities, such as chronic heart disease [7]. This chronic inflammatory condition may significantly influence adropin levels. To our knowledge, this study is the first to investigate changes in adropin levels in patients with newly diagnosed depressive disorders after six months of antidepressant therapy. Most of our patients experienced an increase in adropin levels following the six-month treatment period. However, among those who did not respond to treatment, there was either no change or a slight decrease in adropin levels. This suggests that depression, as an inflammatory disorder, may require a more extended duration of treatment to show significant changes in adropin levels. These changes do not occur suddenly but rather take time to manifest.

Growing evidence shows that depression is associated with chronic inflammation and cell-mediated immune response [22,23,24]. These findings resulted in the search for potential biomarkers of depression diagnosis and treatment. Although numerous studies have investigated molecules such as adropin that would be reliable markers for diagnosing and monitoring depression, such as irisin and preptin [14], no single molecule has been found that would be an exclusive parameter for evaluating depression. Criteria for patients in studies related to depression were not uniform, and they were primarily based on questionnaires to assess depression. Conversely, the patients were selected by completing clinical tests like the Patient Health Questionnaire-9 (PHQ-9) scale and without the exclusion of comorbid psychiatric diagnoses [7,15].

Therefore, the advantage of our study is that depression was diagnosed by a psychiatric expert using BDI-II tests to assess the severity of depression. Our study includes patients who met DSM-5 criteria for a diagnosis of depression and have practically no additional comorbidities such as CHD, diabetes, renal failure, or other chronic diseases that could affect the values of serum adropin.

Regarding various laboratory parameters, baseline measurements showed no significant differences in common laboratory parameters such as leukocytes and CRP in study groups, which is contrary to previous studies [5,7]. Previous studies have investigated various inflammatory markers such as CRP, Interleukin-6 (IL-6), and Tumor Necrosis Factor Alpha (TNF-α). These studies found elevated levels of these markers in patients with depression when compared to healthy controls [25,26]. However, their designs were primarily cross-sectional, with only a single measurement taken during the study. In a similar investigation of depression [27], researchers correlated baseline levels of inflammatory markers (CRP, interleukins, TNF) with the severity of depression and compared their values in response to therapy.

While the majority of the laboratory values under study remained unchanged during follow-up, significant alterations were found in thyroid hormones, GUK, CK, vitamin D, and B12.

We observed a significant increase in vitamin D levels in patients whose symptoms of depression improved after six months of antidepressant therapy. Although the relationship between adropin and vitamin D has not been extensively studied, some evidence suggests that vitamin D may influence the expression or activity of adropin, as both are involved in metabolic processes. Vitamin D is a fat-soluble vitamin essential for bone health, immune function, and various metabolic activities [28,29]. It is produced in the skin in response to sunlight and can also be acquired through certain foods and supplements. A deficiency in vitamin D is characterized by an increase in oxidative stress and pro-inflammatory cytokines, such as IL-1β and IL-6, alongside decreased levels of adropin [30]. Thus, maintaining adequate vitamin D levels may support normal adropin function, which could positively influence metabolism and overall metabolic health. Adropin has been shown to enhance the release of nitric oxide (NO) by stimulating endothelial nitric oxide synthase (eNOS), thereby improving endothelial cell function and promoting neovascularization, ultimately protecting the cardiovascular system [31,32].

We found a significant increase in B12 values in the group of patients in whom the symptoms of depression improved, those who responded positively to therapy with escitalopram. This result is consistent with earlier research on a Finnish population showing a correlation between lower plasma vitamin B12 levels and melancholic depression symptoms [33]. The association between vitamin B12 and depression, as well as whether vitamin B12 supplements can prevent or delay the course of depression, was the subject of numerous studies. According to the findings of one such study, vitamin B12 insufficiency was a significant contributor to the pathophysiology of depression symptoms [34].

According to some studies, teenagers with borderline serum vitamin B12 levels develop cognitive alterations requiring medical treatment [35]. Based on the reviewed studies, it was found that although there is no conclusive evidence showing positive effects of vitamin B12 on depression or depressive symptoms, the lower levels of vitamin B12 in the body are associated with a higher risk of developing depression. In our group of respondents, more women were more susceptible to depression. Moreover, females were identified to be more at risk as compared to men and had coinciding lower serum vitamin B12 levels [36].

## 5. Conclusions

The primary finding is that the responder group’s adropin levels increased throughout the course of the six-month follow-up period of antidepressant treatment, surpassing those of the non-responder group. While the majority of the tested laboratory parameters did not change, significant variations were discovered in vitamin D, B12, and thyroid hormones during follow-up.

To conclude, depression is a complex disease influenced by many factors, including inflammation and immunomodulation. Our study suggests that adropin, among other factors, plays an important role in depression development but also interacts with the depression treatment success. Further investigations to elucidate these pathophysiological mechanisms are needed.

## Figures and Tables

**Table 1:** Baseline characteristics of the depressive disorder (DD) group and the control group. Qualitative variables are presented as numbers (%), and quantitative variables as medians (Q1–Q3; min–max).

	Total	DD Group (N = 54)	Control Group (N = 56)	*p*
Sex				0.848 *
Female	94 (85)	47 (87)	47 (84)	
Male	16 (15)	7 (13)	9 (16)	
Age (Years)	45.5 (31–55; 19–65)	45 (29–57; 19–63)	45.5 (31–53; 21–65)	0.634 **
BMI (kg/m^2^)	23.7 (22–26; 18–40)	23.5 (21–26; 18–40)	23.8 (22–26; 19–31)	0.460 **
Addictions				0.349 *
No	63 (57.3)	28 (51.9)	35 (62.5)	
Yes	47 (42.7)	26 (48.1)	21 (37.5)	
Smoking	43 (39.1)	22 (40.7)	21 (37.5)	
Alcohol Use	1 (1.09)	1 (1.9)	0	
Psychostimulants	1 (1.09)	1 (1.9)	0	
Smoking and Alcohol	2 (1.8)	2 (3.7)	0	
Menopause				0.383 *
No	65 (68)	29 (63)	36 (73)	
Yes	30 (32)	17 (37)	13 (27)	
BDI-II				
Non-depressed 0	56 (50.9)	0	56 (100)	
Mild-depressed 1	13 (11.8)	13 (24.1)	0	
Moderate-depressed 2	12 (10.9)	12 (22.2)	0	
Severe-depressed 3	29 (26.4)	29 (53.7)	0	

Abbreviations: DD—depressive disorder; BMI—body mass index; BDI-II—Beck Depression Inventory; * χ2 test; ** Mann–Whitney test.

**Table 2:** Laboratory parameters of the depressive disorder (DD) group and the control group (data are presented as medians (Q1–Q3; min–max).

Parameter	DD Group (N = 54)	Control Group (N = 56)	*p* *
Leukocytes (×10^9^/L)	6.9 (6–8; 3.7–9.7)	5.9 (5–8; 3.7–11.4)	0.848
GUP (mmol/L)	5.1 (5–6; 2.8–7.2)	5.1 (5–6; 4–6.7)	0.281
CK (U/L)	90 (76–117; 44–481)	87.5 (57–112; 41–279)	0.669
CRP (mg/L)	1.4 (0.7–2; 0.6–26)	0.9 (0.6–1.5; 0.6–5.9)	0.061
Total Cholesterol (mmol/L)	4.9 (4.4–6; 0–8.6)	5.3 (4.7–6; 3.9–8.3)	0.683
Triglycerides (mmol/L)	1.1 (0.6–1.4; 0–3.3)	1 (0.8–1.4; 0.4–5.8)	0.497
HDL Cholesterol (mmol/L)	1.7 (1.3–2.1; 0–2.3)	1.7 (1.3–1.9; 1–2.4)	0.880
LDL Cholesterol (mmol/L)	2.8 (2.4–3.8; 0–6.4)	3.2 (2.5–3.9; 1.6–5.7)	0.510
Fe (μmol/L)	16 (11–21; 5–44)	18 (14–22; 7–29)	0.408
UIBC (μmol/L)	46 (41–54; 24–89)	39 (34–43; 34–56)	0.090
TIBC (μmol/L)	63 (59–69; 44–96)	57.5 (53–62; 45–72)	0.246
TS %	25 (18–34; 6–65)	32 (26–37; 11–49)	0.078
Vitamin B12 (pmol/L)	393 (280–468; 182–760)	367 (309–447; 168–705)	0.204
Folic Acid (nmol/L)	17.4 (14–21; 7.7–45.4)	16.5 (11–20; 5–51)	0.088
Vitamin D (nmol/L)	51.7 (35–65; 18–223)	49.8 (38–63; 24.8–90)	0.911
TSH (mIU/L)	1.17 (0–1.9; 0–4.1)	1.4 (0.27–1.8; 0–3.6)	0.314
T4 (nmol/L)	93 (79–108; 2.3–125)	95 (89–106; 69–127)	0.940
T3 (nmol/L)	1.8 (1.4–2.1; 96–35)	1.5 (1.4–1.6; 1.1–2.1)	0.013
Adropin (ng/mL)	3 (2.5–3.5; 1.4–5.1)	3.1 (2.7–3.6; 0.85–14.8)	0.359

Abbreviations: GUP—glucose; CK—creatine kinase; CRP—C-reactive protein; HDL—high-density lipoprotein; LDL—low-density lipoprotein; Fe—iron; UIBC—unsaturated iron binding capacity; TIBC—total iron binding capacity; TS—transferrin saturation; TSH—thyroid stimulating hormone; T4—thyroxine; T3—triiodothyronine; * χ2 test.

**Table 3:** Beck Depression Inventory (BDI-II): baseline and follow-up in the DD Group.

	BDI-II Baseline			
	Mildly Depressed 1	Moderately Depressed 2	Severely Depressed 3	Total
BDI-II 6-Month Follow-Up				
Non-depressed 0	11	6	15	32
Mildly depressed 1	1	2	6	9
Moderately depressed 2	1	1	5	7
Severely depressed 3	0	0	2	2
Total	13	9	28	50

Abbreviations: Beck Depression Inventory II—BDI-II, light gray: responders, dark gray: non-responders.

**Table 4:** Laboratory parameters of the depressive disorder group at the beginning of the study (baseline) and following six months of treatment. Data are presented as medians (Q1–Q3; min–max).

	Baseline	6-Month Follow-Up	Difference (Baseline—6-Month Follow-Up)	*p* *
Leukocytes (×10^9^/L)	6.4 (5.2–8.1; 3.5–9.9)	6.0 (5.1–7.1; 3.2–10.9)	0.35 (−0.50 to 1.5; −2.3 to 8.7)	0.077
GUP (mmol/L)	5.1 (4.7–5.6; 2.8–8.1)	4.9 (4.6–5.4; 3.9–11.3)	0.150 (−0.3 to 0.5; −3.2 to 6.9)	0.788
CK (U/L)	86 (73–108; 44–481)	87.5 (67–110; 34–964)	5.5 (−11 to 27; −488 to 333)	0.830
CRP (mg/L)	1.1 (0.7–2; 0.6–25)	1.1 (0.6–1.5; 0.6–7.7)	0.10 (−01 to 0.9; −4.3 to 24)	0.216
Total Cholesterol (mmol/L)	4.9 (4.4–6.2; 3.4–8.6)	5.1 (4.2–6.3; 2.9–8.4)	−0.55 (−4.3 to 0.2; −8.4 to 6.2)	0.532
Triglycerides (mmol/L)	0.9 (0.6–1.2; 0.3–3.3)	1.1 (0.7–1.6; 0.4–4.4)	−0.5 (−1 to 0.1; −4.2 to 1)	0.147
HDL Cholesterol (mmol/L)	1.6 (1.3–2.1; 0.9–2.8)	1.5 (1.3–1.9; 0.7–2.9)	−0.1 (−1.2 to 0.1; −2.1 to 2.8)	0.362
LDL Cholesterol (mmol/L)	3 (2.4–3.7; 1.1–6.4)	2.9 (2,2-4;1.1-5.9)	−0.35 (−2.6 to 0.2; −5 to 3.3)	0.776
Fe (μmol/L)	16 (12–20; 5–44)	16 (12–20; 3–67)	1.5 (−6 to 8; −38 to 30)	0.899
UIBC (μmol/L)	45 (39–49; 18–89)	42 (35–47; 11–70)	1.5 (−3 to 10; −21 to 54)	0.332
TIBC (μmol/L)	61 (55–65; 44–96)	58.5 (55–66; 44–96)	0 (−3 to 7; −12 to 64)	0.650
TS %	27 (21–34; 6–65)	30 (22–33; 4–86)	0 (−11 to 9; −59 to 47)	0.754
Vitamin B12 (pmol/L)	377.5 (272–463; 182–760)	381 (312–425; 192–760)	−12 (−67 to 52; −151 to 488)	0.184
Folic Acid (nmol/L)	16.8 (12.8–22.8; 7.7–45.4)	17.8 (11–23.2:5–45)	1.7 (−3.6 to 5.2; −34 to 22)	0.315
Vitamin D (nmol/L)	52.3 (39.8–66; 18–223)	60 (47–69; 19–223)	−7.7 (−20 to 0.12; −61 to 68)	0.034
TSH (mIU/L)	1.24 (0–1.96; 0–4)	1.49 (0.5–2; 0–5.6)	0 (−0.55 to 0.17; −2.6 to 1.6)	0.171
T4 (nmol/L)	95.6 (84–114; 2.3–209)	97.7 (87–107; 74–150)	0.85 (−8.9 to 12.8; −95 to 117)	0.849
T3 (nmol/L)	1.17 (1.4–2.13; 0.96–35)	1.6 (1.5–1.9; 0.93–2.56)	0.09 (−0.16 to 0.3; −0.7 to 33.5)	0.218
Adropin (ng/mL)	3.03 (2.6–3.5; 1.4–5.15)	3.2 (2.7–3.7; 1.5–13.6)	−0.17 (−0.55 to 0.19; −10.7 to 3.1	0.018

Abbreviations: GUP—glucose; CK—creatine kinase; CRP—C-reactive protein; HDL—high-density lipoprotein; LDL—low-density lipoprotein; Fe—iron; UIBC—unsaturated iron binding capacity; TIBC—total iron binding capacity; TS—transferrin saturation; TSH—thyroid stimulating hormone; T4—thyroxine; T3—triiodothyronine; * Wilcoxon test.

**Table 5:** The analysis of changes in laboratory parameters during the six-month follow-up in responders and non-responders. Data are presented as medians (Q1–Q3; min–max).

	Responders (N = 32)		*p* *	Non-Responders (N = 18)		*p* *
	Baseline	6-Month Follow-Up		First Analysis	6 Months Later Analysis	
Leukocytes (×10^9^/L)	6.5 (4.7–8.2; 3.7–9.9)	5.9 (5–7.5; 3.8–11)	0.266	6.4 (6–7.9; 3.5–9.9)	6.1 (5.4–6.5; 3.2–8.1)	0.155
GUP (mmol/L)	5.1 (5.1–4.7; 2.8–8.1)	5.1 (4.7–5.4; 4–11)	0.650	5 (4.7–5.4; 3.9–7.2)	4.7 (4.4–5.6; 3.9–7.2)	0.206
CK (U/L)	81 (65–94; 44–481)	90 (70–101; 34–964)	0.362	90 (79–126; 52–288)	84 (65–117; 47–270)	0.309
CRP (mg/L)	1.45 (0.6–2; 0.6–25)	1.1 (0.6–1.8; 0.6–7.7)	0.345	1.05 (0.7–1.9; 0.6–5.5)	1.05 (0.6–1.4; 0.6–3.9)	0.306
Total Cholesterol (mmol/L)	5.2 (4.7–6.3; 3.5–8.6)	5.2 (4.2–6.4; 3.4–8.1)	0.777	4.7 (4.4–5.9; 3.4–6.8)	5.1 (4–5.9; 2.9–8.4)	0.553
Triglycerides (mmol/L)	1 (0.5–1.7; 0.5–3.3)	1 (0.7–1.6; 0.4–4.4)	0.206	0.9 (0.7–1.1; 0.3–1.7)	1.3 (0.8–1.4; 0.4–3.1)	0.443
HDL Cholesterol (mmol/L)	1.6 (1.3–2.1; 0.9–2.2)	1.5 (1.3–1.8; 0.7–2.4)	0.328	1.6 (1.2–2; 1–2.8)	1.6 (1.2–2; 0.9–2.9)	0.951
LDL Cholesterol (mmol/L)	3 (2.5–3.8; 1.1–6.4)	3.1 (2.2–4; 1.7–5.9)	0.856	2.9 (2–3.3; 1.2–4.7)	2.8 (1.8–3.7; 1.1–4.9)	0.722
Fe (μmol/L)	16 (12–21; 5–30)	15.5 (12–20; 3–43)	0.969	16 (11–19; 7–44)	16.5 (11–20; 7–67)	0.623
UIBC (μmol/L)	43.5 (36–49; 18–73)	40 (33–45; 20–70)	0.394	45 (39–50; 24–89)	45 (41–51; 11–68)	0.670
TIBC (μmol/L)	59.5 (55–64; 44–80)	57 (52–64; 44–73)	0.345	64 (56–67; 51–96)	61 (56–69; 49–96)	0.531
TS %	27.5 (20–35; 6–62)	30 (23–32; 4–66)	0.903	25 (21–31; 7–65)	27 (19–36; 11–86)	0.641
Vitamin B12 (pmol/L)	334 (225–415; 204–668)	372 (311–409; 192–546)	0.085	413 (339–488; 182–760)	405 (316–433; 206–760)	0.943
Folic Acid (nmol/L)	15.5 (12–22; 8–32)	17.8 (11–22; 6–45)	0.688	17.7 (14–23; 9–45)	17.8 (12–24; 5–37)	0.246
Vitamin D (nmol/L)	54 (38–66; 18–106)	63.6 (43–80; 19–110)	0.102	50 (43–68; 30–223)	59 (48–71; 28–223)	0.227
TSH (mIU/L)	1.1 (0–1.5; 0–3.9)	1.5 (0–1.9; 0–5.6)	0.042	1.8 (0.5–2.1; 0–4.1)	1.8 (1.2–2.3; 0–3.7)	0.672
T4 (nmol/L)	95 (84–118; 21–130)	98 (88–107; 74–150)	0.969	96 (85–108; 23–209)	97 (87–107; 75–143)	0.925
T3 (nmol/L)	1.7 (1.4–2; 0.96–32)	1.6 (1.5–1.7; 0.93–2.4)	0.405	1.8 (1.6–2.1; 1.1–35)	1.8 (1.5–2; 1.1–2.5)	0.301
Adropin (ng/mL)	3.07 (2.55–3.53; 1.4–4.8)	3.36 (2.97–3.84; 1.48–5.71)	0.001 *	2.86 (2.44–3.34; 1.90–5.15)	2.88 (2.41–3.36; 2.1–13.67)	0.903

Abbreviations: GUP—glucose; CK—creatine kinase; CRP—C-reactive protein; HDL—high-density lipoprotein cholesterol; LDL—low-density lipoprotein; Fe—iron; UIBC—unsaturated iron binding capacity; TIBC—total iron binding capacity; TS—transferrin saturation; TSH—thyroid stimulating hormone; T4—thyroxine; T3—triiodothyronine; * Wilcoxon test.

**Table 6:** Changes in laboratory parameters after the treatment of the depression disorder group between responders vs. non-responders.

	Differences Between Baseline and 6-Month Follow-Up Median (Q1–Q3; Min–Max)		
	Responders (N = 32)	Non-Responders (N = 18)	*p* *
Leukocytes (×10^9^/L)	0.1 (−0.55 to 1; 2.3 to 3.4)	0.5 (−0.5 to 2.7; −1.3 to 8.7)	0.192
GUP (mmol/L)	0.05 (−0.55 to 0.4; −3.2 to 1.6)	0.25 (0 to 0.9; −1.2 to 6.9)	0.045
CK (U/L)	−2.5 (−14 to 9; −488 to 333)	18 (5 to 36; −137 to 126)	0.027
CRP (mg/L)	0 (−0.25 to 0.65; −4.3 to 23.9)	0.25 (0 to 1; −1.3 to 5.5)	0.273
Total Cholesterol (mmol/L)	−0.6 (−4.3 to 0.15; −6.9 to 2.5)	−0.45 (−4.2 to 0.2; −8.4 to 6.2)	0.679
Triglycerides (mmol/L)	−0.6 (−0.95 to 0.05; −4.2 to 1)	−0.3 (−1.2 to 0.2; −3.1 to 0.9)	0.422
HDL Cholesterol (mmol/L)	−0.15 (−1.2 to 0.15; −2.1 to 0.5)	−0.1 (−1.2 to 0.1; −2.1 to 2.8)	0.778
LDL Cholesterol (mmol/L)	−0.8 (−2.6 to 0.1; −5 to 2)	−0.15 (−1.4 to 0.2; −4.9 to 3.3)	0.283
Fe (μmol/L)	2 (−7 to 7; −38 to 14)	0.5 (−4 to 9; −31 to 30)	0.503
UIBC (μmol/L)	2.5 (−4.5 to 8; −21 to 50)	0.5 (−3 to 13; −14 to 54)	0.591
TIBC (μmol/L)	0.5 (−2 to 7; −12 to 12)	0 (−3 to 6; −10 to 64)	0.888
TS %	2.5 (−13 to 9; 159 to 31)	0 (−5 to 13; −33 to 47)	0.672
Vitamin B12 (pmol/L)	−32.5 (−70 to 14.4; −135 to 275)	21 (−48 to 144; −151 to 488)	0.032
Folic Acid (nmol/L)	0.88 (−4.5 to 3.9; −34 to 13)	1.8 (−0.8 to 14; −19.2 to 22.2)	0.162
Vitamin D (nmol/L)	−3.9 (−29 to 11; −49 to 40)	−5.6 (−13.3 to 26.1; −61 to 68)	0.303
TSH (mIU/L)	−0.21 (−0.98 to 0.002; −2.6 to 1.2)	0 (−0.16 to 0.33; −2.4 to 1.6)	0.097
T4 (nmol/L)	−0.05 (−9.5 to 10; −95 to 31)	3.5 (−6 to 16.1; −79 to 117)	0.388
T3 (nmol/L)	0.085 (−0.18 to 0.25; −0.47 to 31)	0.19 (−0.11 to 1.25; −0.78 to 33.5)	0.202
Adropin (ng/mL)	−0.175 (−0.55 to 0.040; −1.58 to 1.29)	0.21 (−0.43 to 1.05; −10.68 to 3.11)	0.048

Abbreviations: GUP—glucose; CK—creatine kinase; CRP—C reactive protein; HDL—high-density lipoprotein; LDL—low-density lipoprotein; Fe—iron; UIBC—unsaturated iron binding capacity; TIBC—total iron binding capacity; TS—transferrin saturation; TSH—thyroid stimulating hormone; T4—thyroxine; T3—triiodothyronine; * Mann–Whitney test.

## Data Availability

The data presented in this study are available on request from the corresponding author due to privacy and ethical reasons. Raw data can be found at corresponding author via e-mail: lovric.sanja@gmail.com.

## Abbreviations

The following abbreviations are used in this manuscript:

Akt/GSK3β	serine/threonine kinase glycogen synthase kinase 3β
BDI II	Beck Depression Inventory-II
BMI	body mass index
BDNF	brain-derived neurotrophic factor
CHD	coronary heart disease
CK	creatine kinase
CRP	C-reactive protein
CNS	central nervous system
DDG	depressive disorder group
DSM-5	Diagnostic and Statistical Manual of Mental Disorders, Fifth Edition
EDTA	Ethylenediaminetetraacetic acid
Enho	Energy homeostasis-associated gene
eNOS	endothelial nitric oxide synthase
ERK ½	extracellular signal-regulated kinases 1/2
LDL-C	low-density lipoprotein cholesterol
NO	nitric oxide
PHQ-9	Patient Health Questionnaire-9
SCID	Structured Clinical Interview for Diagnostic
SPSS	Statistical Package for the Social Sciences
TIBC	total iron binding capacity
TSH	thyroid-stimulating hormone
TNFα	tumor Necrosis Factor Alpha
UIBC	unsaturated iron binding capacity
VEGFR-2	vascular endothelial growth factor receptor-2
